# Carbapenem-resistant Acinetobacter baumannii bloodstream infections and specific phages isolation, analysis and application

**DOI:** 10.3389/fcimb.2026.1774993

**Published:** 2026-04-01

**Authors:** Lu Wang, Fuhua Wang, Zhiyong Yuan, Ying Liu, Yajun Jing, Jinyan Xing

**Affiliations:** Department of Critical Care Medicine, The Affiliated Hospital of Qingdao University, Qingdao, Shandong, China

**Keywords:** bacteriophages, bloodstream infection, carbapenem-resistant Acinetobacter baumannii, multi-drug resistance, vB_AbaP_CV1

## Abstract

**Introduction:**

Antimicrobial resistance poses a major challenge in the treatment of *A. baumannii* worldwide, especially *Carbapenem-Resistant A. baumannii* (CRAB) bloodstream infections.

**Objectives:**

The objective of this study was to isolate and characterize a CRAB-targeting bacteriophage and to evaluate its therapeutic potential, alone and in combination with polymyxin B.

**Methods:**

From January 2020 to September 2025, adult patients with *A. baumannii* bloodstream infection were enrolled. Clinically relevant data were collected. *A. baumannii* strains were isolated from clinical samples and the phage was isolated from wastewater samples collected from hospital by double-layer agar plate method. The synergistic activity of phage–polymyxin B combination therapy was assessed by checkerboard analysis and time-kill assays. BALB/c mice were infected with a CRAB suspension via tail vein to establish the model and were subsequently treated with the phage and phage-antibiotic combination.

**Results:**

A total of 50 patients suffered from bloodstream infections caused by Acinetobacter baumannii. Among them, 34 (68%) cases were classified as CRAB. Compared with CSAB, they underwent a longer duration of mechanical ventilation(13.00(6.00,28.00) vs.3.00(2.00,4.00),P =0.019), used more triple therapy(9.41% vs.0%,P=0.041), and had a higher in-hospital mortality(82.35% vs.18.75%,P <0.001). Synergistic antibacterial activity between the phage and colistin B was demonstrated using the checkerboard assay and time-kill curve analysis. In a murine bacteremia model, the vB_AbaP_CV1-antibiotic combination significantly reduced tissue bacterial loads, attenuated inflammatory responses, and ameliorated clinical manifestations. Notably, the combined therapy exhibited superior therapeutic efficacy compared to either monotherapy alone.

**Conclusion:**

CRAB bloodstream infections are associated with high mortality and poor outcomes. vB_AbaP_CV1 can lyse the CRAB strains. Both phage monotherapy and the phage-colistin B combination exhibited therapeutic efficacy, with the combined regimen yielding the optimal outcome.

## 1 Introduction

*A. baumannii* is a key nosocomial pathogen capable of acquiring novel antibiotic resistance genes, evading therapeutic agents Recently, the resistance rate of *A. baumannii* to all commonly used antimicrobials has increased rapidly, with a large proportion being carbapenem-resistant Acinetobacter baumannii (CRAB) (Tomczyk et al., 2019). It has now become one of the important nosocomial pathogens, particularly in the ICU, where it is responsible for a wide range of nosocomial infections, including ventilator-associated pneumonia (VAP), central line-associated bloodstream infections (CLABSI), and urinary tract infections (UTIs). It has been associated with an estimated 326,000 global deaths in 2019 (Iovleva et al., 2025). Prevalence of multidrug-resistant *A. baumannii* (MDRAB) in patients with nosocomial pneumonia has been reported to range between 40 to 95% (Gordon and Wareham, 2010; Ziółkowski et al., 2018), and its associated mortality has been reported to range between 45 to 85% (Zheng et al., 2013; Ozgur et al., 2014).

Bacteremia can arise through dissemination from an infection, commonly pneumonia or urinary tract infections (UTIs), or through contaminated medical devices (Holmes et al., 2021). Once in the bloodstream, CRAB can rapidly lead to systemic complications such as sepsis, septic shock, and multi-organ failure. The prevalence of carbapenem resistance and carbapenem-colistin dual resistance in Gram-negative blood culture isolates from patients with bloodstream infections is unacceptably high. Patients with bloodstream infections due to carbapenem-resistant isolates had substantially higher mortality (Balkhair et al., 2023). In a recent study on clinical outcomes of CRAB infection, bloodstream infection was independently associated with 30-day mortality (Wang et al., 2024).

However, the options for treating *A. baumannii* infections are severely limited. Inherent and acquired resistance mechanisms, as well as host factors, significantly restrict options for empirical medication (Zhang et al., 2024). Multiple authoritative guidelines, including the European Society of Clinical Microbiology and Infectious Diseases (ESCMID) and the Infectious Diseases Society of America (IDSA) antimicrobial resistance (AMR) treatment guidelines, recommend selecting at least two antibiotics for anti-infective therapy when treating patients with severe CRAB infections (Paul et al., 2022; Tamma et al., 2022). Moreover, inappropriate drug coverage can have detrimental effects on patients. Phage-based therapy has emerged as a potential treatment option for *A. baumannii* infections.

Bacteriophages are viruses specifically infecting and lysing bacteria. Bacteriophage therapy for treating bacterial infections has a history spanning over 100 years (Dąbrowska and Abedon, 2019). Phages target specific bacterial species by binding to surface receptors on the bacterial cell, injecting their genetic material, and hijacking the bacterial machinery to replicate themselves. They only target the pathogenic bacteria, leaving the normal microbiota intact (Zurabov et al., 2023). Schooley has demonstrated a personalized bacteriophage-based therapeutic treatment for a patient with necrotizing pancreatitis complicated by an MDR *A. baumannii* infection (Schooley et al., 2017). Cha has reported two novel bacteriophages and evaluated their therapeutic efficacy *in vivo* (Cha et al., 2018). However, previous phage therapy studies have primarily focused on Acinetobacter baumannii-induced pneumonia and skin wound infections, with limited research on bloodstream infections, especially those caused by CRAB. There remain significant research gaps in using bacteriophages for treating clinically complex carbapenem-resistant Acinetobacter baumannii (CRAB) infections.

In this study, we reported the incidence and mortality rate of CRAB bloodstream infections in the local area. We also isolated and identified bacteriophages based on local CRAB bloodstream infection samples. Furthermore, we established a model of CRAB bloodstream infection to verify it. We aimed to provide a new and acceptable approach for the treatment of CRAB bloodstream infections and reduce the mortality.

## 2 Method

### 2.1 Patient enrollment and clinical data collection

We conducted a retrospective review of adults (aged ≥18 years) with Acinetobacter baumannii bloodstream infection, confirmed by microbial culture and identification from blood samples, who were hospitalized at the West Coast Campus of the Affiliated Hospital of Qingdao University from January 2020 to September 2025. Patients aged <18 years or with incomplete clinical data were excluded. Baseline demographic characteristics, microbiological data, antimicrobial susceptibility test results, and patient outcomes were retrospectively collected. The study was reviewed and approved by the Ethics Review Committee of The Affiliated Hospital of Qingdao University (No. QYFYWZLL30688).

### 2.2 Isolation, Identification of bacteria strain

An Acinetobacter baumannii strain was isolated from a blood sample of a clinical patient at Qingdao University Affiliated Hospital. Through antibiotic susceptibility testing, it was confirmed to be a multidrug-resistant strain and then designated it as CRAB-1. The strain was cultured on LB agar plates under an atmosphere containing 5% CO_2_ at 37 °C or in fluid medium at 37 °C with agitation. Bacterial genome sequencing was conducted by the Illumina platform. Genomic DNA was extracted from the bacterial cell pellets using the OMEGA Bacteria DNA Kit following the manufacturer’s instructions. The raw paired-end reads were quality trimmed and controlled using Trimmomatic software, and genome assembly was performed using ABySS 2.2.0. Genome annotation was performed using GeneMark for gene prediction, followed by an analysis of the genetic characteristics of the bacterial isolate, including MLST typing based on the assembled genome and drug resistance genes annotated by blasting against the CARD database.

### 2.3 Phage isolation and purification

Phages were isolated from wastewater samples collected from the Affiliated Hospital of Qingdao University. A mixture of wastewater and CRAB-1 bacterial culture (grown overnight with shaking) was incubated at 37 °C with shaking for 24 h to allow for phage proliferation. A double-layer agar method was employed for phage isolation and purification. Specifically, the logarithmic-phase bacterial culture and the phage-enriched solution were mixed and poured onto a semi-solid LB agar medium as the top layer. Independent plaques were selected and transferred to SM buffer, repeating the process 4–5 times until consistent plaque morphology was observed. The purified phage was stored at 4°C for further experiments.

### 2.4 Morphological observation of phage via transmission electron microscopy

Purified and concentrated phage suspension (10^9^ PFU/mL) was adsorbed onto a carbon-coated copper grid, negatively stained with 2% phosphotungstic acid, and air-dried. Observation was carried out using a JEOL JEM-1200EX electron microscopy.

### 2.5 Analysis biological characteristics of the phage

Optimal multiplicity of infection (MOI) was used to determine the optimal interaction ratio between phages and host bacteria. The bacterial suspension (1 × 10^8^ CFU/mL) was mixed with phage at MOI values of 10, 1, 0.1, 0.01 and 0.001. The mixture was cultured with shaking in a shaker at 37 °C and 160 rpm for 2 hours, after which the supernatant was collected by centrifugation and the phage titer was determined by the double-layer agar method. The highest phage titer was regarded as the optimal MOI for the phage.

The environmental stability of phages reflects their viability. Phage lysates were treated at different temperatures (-20 to 80°C) and different pH values (2 to 12) for 1 hour, and the phage titers were determined using the double-layer agar method.

The one-step growth curve of bacteriophages was analyzed to understand their infection dynamics and life cycle. A one-step growth curve was performed to characterize the life cycle of the phage. Host bacteria (1 × 10^8^ CFU/mL) were mixed with the phage at the optimal MOI of 1 and incubated at 37°C for 5 min. After centrifugation, the pellet was resuspended in 10 mL of fresh LB medium and reincubated. Samples of 100 μL were collected every 10 min, and the phage titer was determined using the double-layer agar method.

### 2.5 Host range determination

The host range of the phage was determined using 40 bacterial strains isolated from different clinical patients at different time points, including 25 CRAB strains, 5 *Klebsiella pneumoniae strains*, 5 *Escherichia coli strains*, and 5 *Pseudomonas aeruginosa strains*. The spot test was performed as follows: 100 μL of bacterial culture was mixed with 5 mL of semi-solid LB medium and overlaid onto an LB agar plate. After solidification, 10 μL of phage lysate was spotted onto the plate. Following incubation at 37°C for 24h, the formation of clear lysis plaques was observed.

### 2.6 Phage bioinformatics analysis

According to the instructions, the genomic DNA of the phage were extracted using the OMEGA Viral DNA Extraction Kit. Whole-genome sequencing of DNA was performed on an Illumina HiSeq 6000 platform. Genome information was annotated by kegg, blastp (https://blast.ncbi.nlm.nih.gov). MEGA 11.0 was employed to construct a phylogenetic tree based on the amino acid sequences of DNA polymerase. Virulence genes and antibiotic resistance genes carried by the phage were predicted by alignment against the Virulence Factors Database (VFDB) and the Comprehensive Antibiotic Resistance Database (CARD).

### 2.7 Determination of *in vitro* bactericidal efficiency of phage combined with polymyxin B

The synergistic effect of phage and polymyxin B was determined using the checkerboard assay. In a 96-well plate, two-fold serially diluted polymyxin B was added horizontally, and ten-fold serially diluted phage (10^8^ PFU/mL) was added vertically, followed by the bacterial suspension at a final concentration of 10^5^ CFU/mL. After incubation at 37 °C overnight, the absorbance at 600 nm (OD_600_) was measured, and the fractional inhibitory concentration index (FICI) was calculated.

To confirm phage-antibiotic synergy, a time-kill experiment was employed. Bacterial suspension at a final concentration of 1×10^5^ CFU/mL was inoculated into 96-well plates. The OD_600_ values of the phage monotherapy group, polymyxin B monotherapy group, phage-polymyxin B combination group and control group were measured continuously for 24 h.

### 2.8 Biofilm formation inhibition and formed biofilm removal

Biofilm formation inhibition assay: 500 μL of bacterial suspension at 1×10^5^ CFU/mL was added to 48-well plates, followed by 500 μL of PBS, phage suspension, polymyxin B solution, and a 1:1 mixture of phage and polymyxin B, respectively. The plates were incubated at 37 °C for 24 h.

Mature biofilm eradication assay: 1 mL of bacterial suspension at 1×10^5^ CFU/mL was added to 48-well plates and incubated at 37 °C for 48 h to allow mature biofilm formation. After removing the culture medium, 500 μL of the corresponding treatment solutions was added according to the above groups, followed by incubation at 37 °C for 24 h.

Biofilm quantification was performed using crystal violet staining. After incubation, the culture medium was discarded, and the wells were gently washed three times with PBS and air-dried at room temperature. Then, 1% crystal violet solution was added for staining for 10 min. The stain was removed, the wells were washed with PBS and air-dried, followed by the addition of 95% ethanol for decolorization for 10 min. The absorbance at 595 nm (OD_595_) of each group was measured.

### 2.9 *In vivo* murine model of CRAB bloodstream infection

Specific pathogen-free (SPF) female BALB/c mice (5–7 weeks old, ~20 g) were used to establish a CRAB *in vivo* infection model.

The CRAB-1 strain was cultured in 5 mL LB medium for 10 h, centrifuged at 3000 r/min for 10 min, washed twice with PBS, and resuspended in PBS to adjust concentration. For pre-experiment, 100 μL CRAB suspension (10^5^–10^8^ CFU/mL) was tail-vein injected (n=8); mice were observed for 7 days, and 10^7^ CFU/mL was determined as the formal infection dose. In formal experiment, infected mice were randomly divided into 4 groups (n=8). At 2 h post-infection, treatments were: (i) PBS; (ii) 10^9^ PFU/mL phage; (iii) 5 mg/kg polymyxin B (PB); (iv) 10^9^ PFU/mL phage + 5 mg/kg PB. A non-infected blank control group was set.

The experiment lasted 48 h, with survival and MSS clinical scores recorded. At 48 h post-treatment, mice were anesthetized with 2%–3% isoflurane for 2–3 min via inhalation induction. Subsequently, peripheral blood was collected by enucleation for bacterial load quantification and the detection of serum inflammatory factors (TNF-α, IL-1β, IL-6). The mice were then euthanized by cervical dislocation, followed by sterile collection and weighing of the kidneys, liver, lungs, and spleen. A portion of the tissue samples was homogenized for CFU quantification, and the remaining tissues were fixed in 4% paraformaldehyde for H&E staining and pathological observation.

All experiments complied with NIH Publication No. 85-23 (revised 1996) and were approved by the Animal Care and Welfare Committee of the Affiliated Hospital of Qingdao University (Ethical Approval No.QYFYWZLL30688).

### 2.10 Statistic analysis

Normally distributed quantitative data are presented as mean ± standard deviation, and non-normally distributed data as median (interquartile range). Intergroup comparisons were performed using the Chi-square test for qualitative variables, the two-sample t-test for normally distributed quantitative variables, and the Mann-Whitney U test for non-normally distributed variables. A two-sided p-value of less than 0.05 was considered statistically significant. The statistical analysis was performed using SPSS software (version 22.0; SPSS, Inc., Chicago, IL, USA).

## 3 Result

### 3.1 Characteristics of patients with CRAB bloodstream infections

A total of 54 patients were initially identified during the study period. After applying the inclusion and exclusion criteria, 50 patients were included in the final analysis. A total of 50 patients were included and divided into carbapenem - resistant Acinetobacter baumannii (CRAB) blood culture - positive group and carbapenem - susceptible Acinetobacter baumannii (CSAB) blood culture - positive group according to the results of drug susceptibility tests. Among them, 34/50 (68%) were CRAB. The basic characteristics of patients with Acinetobacter baumannii are shown in **Table 1**.

**Table 1:** Characteristics of patients with *A. baumannii* infection.

Variables	Total (n = 50)	CSAB (n = 16)	CRAB (n = 34)	*P*
Demographic characteristics				
Age (years)​	62.34 ± 11.70	62.56 ± 11.41	62.24 ± 12.00	0.928
Male	37 (74.00)	14 (87.50)	23 (67.65)	0.251
Baseline health status				
Hypertension	22 (44.00)	6 (37.50)	16 (47.06)	0.525
Diabetes	20 (40.00)	6 (37.50)	14 (41.18)	0.804
Liver Insufficient	17 (34.00)	3 (18.75)	14 (41.18)	0.118
Renal Insufficient	18 (36.00)	4 (25.00)	14 (41.18)	0.266
Malignant Tumor	14 (28.00)	9 (56.25)	5 (14.71)	**0.007**
Cardiac Insufficient	22 (44.00)	6 (37.50)	16 (47.06)	0.525
Multiple Organ Failure	31 (62.00)	2 (12.50)	29 (85.29)	**<.001**
Hormone/Immunosuppressant Use	26 (52.00)	1 (6.25)	25 (73.53)	**<.001**
APACHii score	17.74 ± 7.58	12.88 ± 5.44	20.03 ± 7.42	**0.001**
Invasive procedures and catheter use				
Mechanical Ventilation	37 (74.00)	6 (37.50)	31 (91.18)	**<.001**
Duration of Mechanical Ventilation	12.00 (5.00, 27.00)	3.00 (2.00, 4.00)	13.00 (6.00, 28.00)	**0.019**
Deep Venous Catheterization	35 (70.00)	4 (25.00)	31 (91.18)	**<.001**
Urinary Catheter	39 (78.00)	8 (50.00)	31 (91.18)	**0.004**
CRRT	20 (40.00)	2 (12.50)	18 (52.94)	**0.006**
Microbial infection status				
Bacterial Co-infection (Non-BSI)	30 (60.00)	6 (37.50)	24 (70.59)	**0.026**
Fungal Co-infection (Non-BSI)	22 (44.00)	1 (6.25)	21 (61.76)	**<.001**
Viral Co-infection (Non-BSI)	9 (18.00)	0 (0.00)	9 (26.47)	0.06
Treatment				
Dual antibiotic therapy	21 (42.00)	4 (25.00)	17 (50.00)	0.095
Triple antibiotic therapy	10 (20.00)	0 (0.00)	10 (29.41)	**0.041**
Carbapenems	33 (66.00)	2 (12.50)	31 (91.18)	**<.001**
Sulbactam and Its Combinations Use	15 (30.00)	7 (43.75)	8 (23.53)	0.261
Tigecycline Use	22 (44.00)	0 (0.00)	22 (64.71)	**<.001**
Polymyxin B Use	14 (28.00)	0 (0.00)	14 (41.18)	**0.007**
Outcomes				
In-hospital Death	31 (62.00)	3 (18.75)	28 (82.35)	**<.001**

Bold values indicate statistically significant differences between the CSAB and CRAB groups (P < 0.05).

A greater proportion of patients with CRAB infections developed multiple organ dysfunction syndrome (MODS), defined as the concurrent dysfunction of two or more organ systems. These patients also had a higher prevalence of immunosuppression, including corticosteroid therapy. They underwent a greater number of invasive procedures, including central venous catheterization, mechanical ventilation, urinary catheterization and CRRT. The duration of mechanical ventilation was significantly longer in patients with CRAB infections,(13.00(6.00,28.00) vs.3.00(2.00,4.00),P =0.019). A higher proportion of concurrent bacterial and fungal infections was observed in patients with CRAB infection compared to those with CSAB infection. In terms of treatment, the use of triple therapy (consisting of a carbapenem, tigecycline, and colistin) was more common in patients with CRAB infection than in those with CSAB infection(29.41% vs.0%,P=0.041).Patients with CRAB infection had a significantly higher in-hospital mortality rate than those with CSAB infection (82.35% vs.18.75%,P <0.001). Shown in **Table 1**.

### 3.2 Phage vB_AbaP_CV1 isolation and morphology

CRAB-1 was used as the host strain for phage isolation. Whole-genome sequencing and bioinformatics analysis showed that this strain carries the *blaOXA-23* gene encoding the intrinsic carbapenemase. (**Supplementary Table 1**) Multilocus sequence typing (MLST) identified the strain as ST369. Antimicrobial susceptibility testing based on the latest CLSI guidelines confirmed that CRAB-1 is susceptible to polymyxin B and tigecycline (**Supplementary Table 2**).

**Figure 1:**
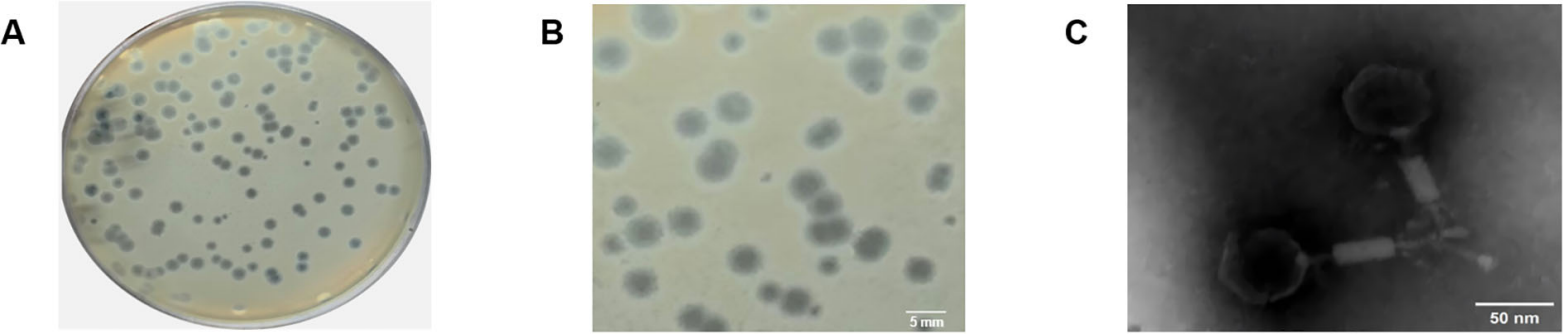
Morphology of phage vB_AbaP_CV1 **(A)** Plaques formed by vB_AbaP_CV1 on double-layer agar plates; **(B)** Detailed view of vB_AbaP_CV1 plaques; **(C)** TEM image of vB_AbaP_CV1.

Using CRAB-1 as the host, a lytic phage designated vB_AbaP_CV1 was isolated from sewage samples. This phage produced transparent plaques of 4.0–6.0 mm in diameter on LB agar plates, with frequent plaque overlap (**Figures 1A, B**). Transmission electron microscopy (TEM) showed that vB_AbaP_CV1 has an icosahedral head with a side length of approximately 30 nm, a rigid tail of about 70 nm in length and 15 nm in diameter, and tail fibers approximately 10 nm long (**Figure 1C**).

The basic physiological characteristics of vB_AbaP_CV1 are shown in **Supplementary Figure 1**.

### 3.3 Biological characteristics of vB_AbaP_CV1

The host range of the vB_AbaP_CV1 was determined using 25 clinically isolated CRAB strains derived from different times and different patients. The results showed that the vB_AbaP_CV1 exhibited lytic activity against 18 out of the 25 tested CRAB strains. No lytic activity was observed against *K. pneumoniae*, *E. coli*, or *P. aeruginosa* (**Supplementary Table 3**).

**Figure 2:**
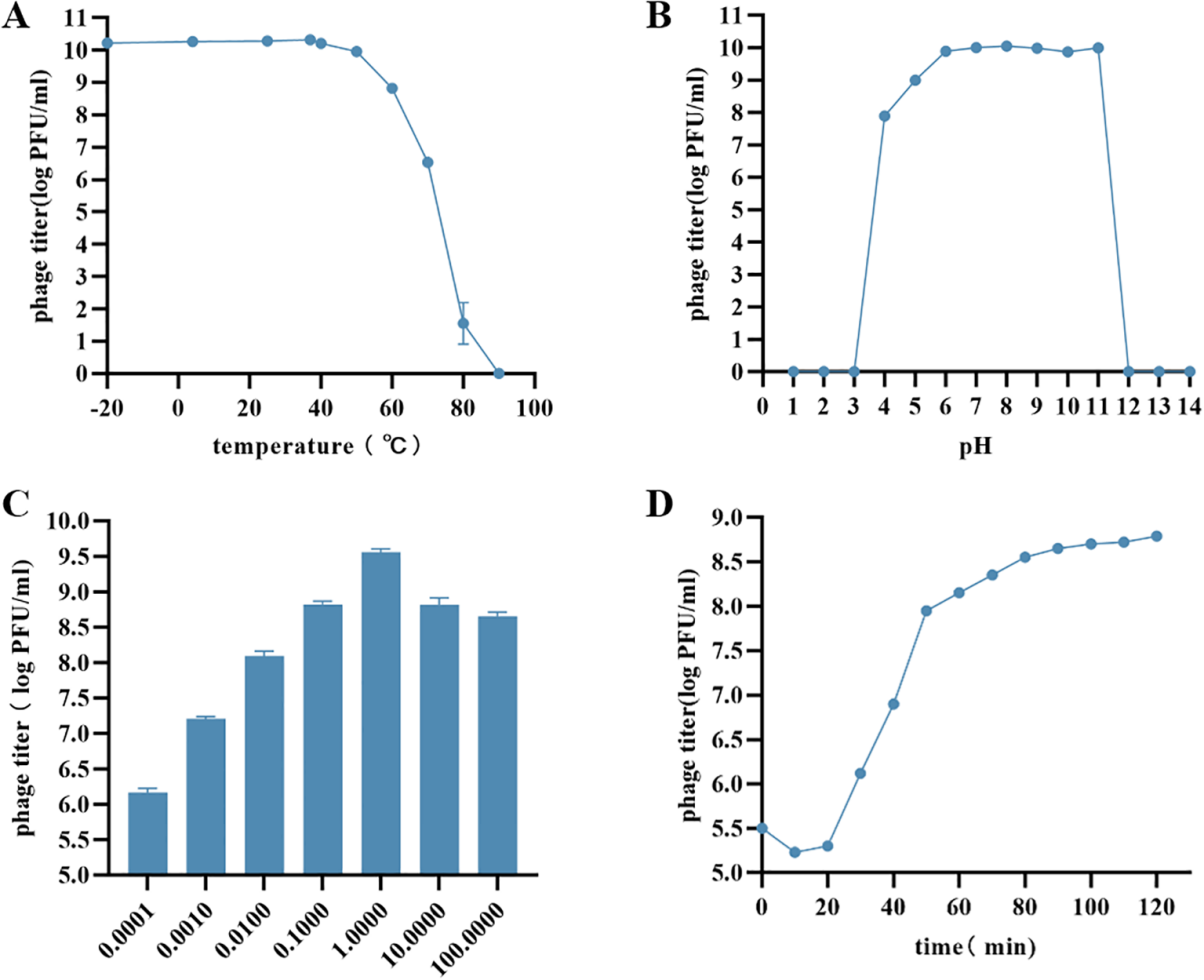
Biological characters of vB_AbaP_CV1. **(A)** Temperature stability; **(B)** pH stability; **(C)** Multiplicity of infection; **(D)** One step-growth curve.

The vB_AbaP_CV1 maintained stable lytic activity from -20 °C to 60 °C. At 70 °C, the phage titer decreased by 10^4^ PFU, and the phage was almost completely inactivated at 80 °C (**Figure 2A**). The phage retained good activity over a pH range of 4 to 11 (**Figure 2B**). The optimal multiplicity of infection (MOI) was defined as the ratio of phage to bacterium that produced the maximum number of progeny phages. As shown in the figure, the optimal MOI of the phage was 1 (**Figure 2C**). One-step growth curve analysis revealed the growth characteristics of the phage, with a latent period of 20 min (**Figure 2D**).

### 3.4 Whole genome characterization of vB_AbaP_CV1

**Figure 3:**
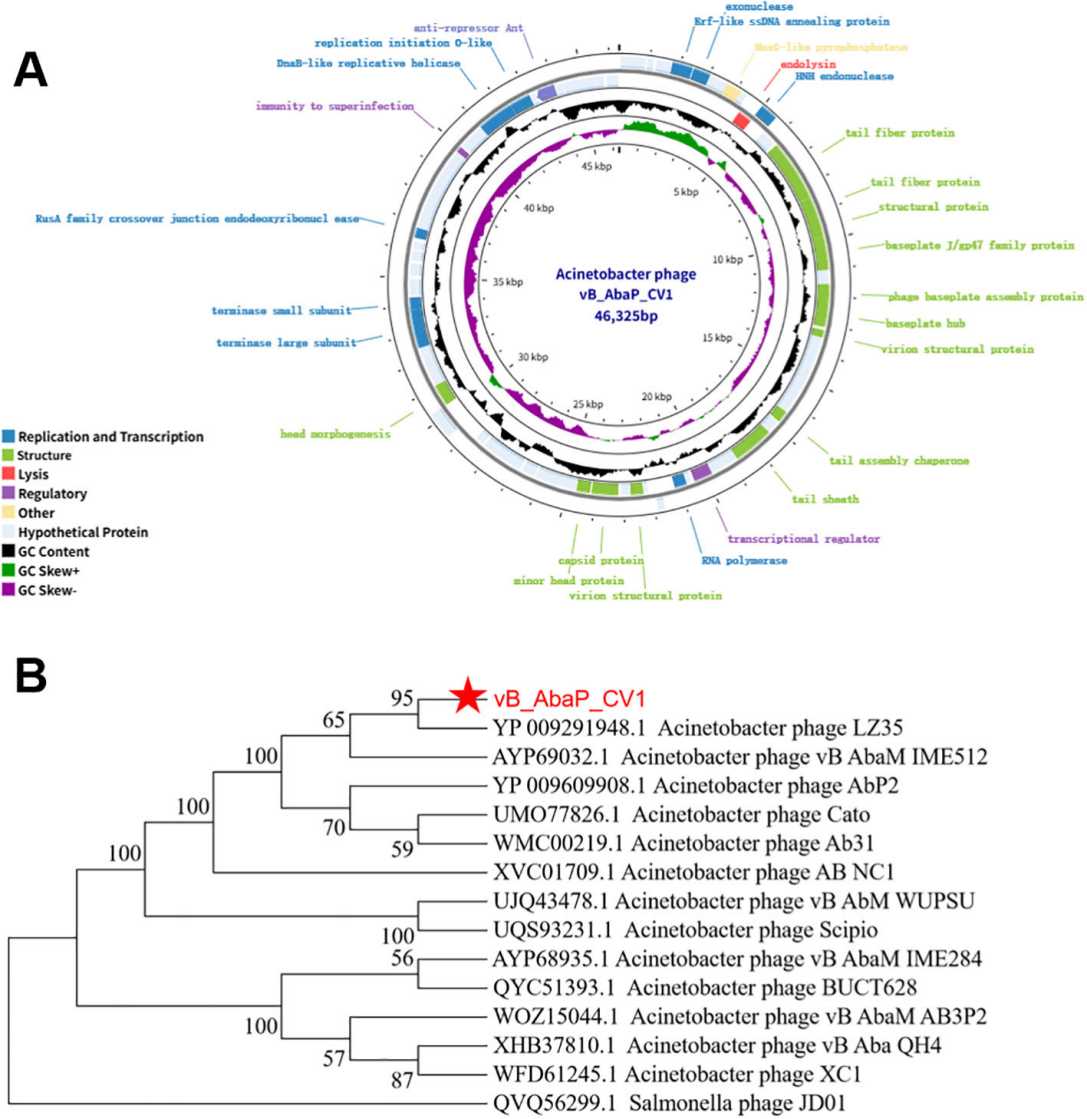
Genomic features of vB_AbaP_CV1. **(A)** Genomic map of phage vB_AbaP_CV1. The innermost circle represents GC skew (+, green; -, purple), and the middle black circle represents GC content. The two outermost circles show the predicted open reading frames (ORFs) of the phage. Coding sequences (CDSs) in different colors represent different functional categories: nucleic acid replication and transcription (blue); structural related proteins (green); host lysis proteins (red); regulatory proteins (purple); hypothetical proteins (gray). **(B)** Phylogenetic analysis of phage vB_AbaP_CV1based on the terminase large subunit gene sequence, constructed using the maximum likelihood method. Bootstrap values are shown on the nodes to indicate the robustness of the phylogenetic relationships. The scale bar represents the number of amino acid substitutions per site.

The genome of phage vB_AbaP_CV1 consists of double-stranded DNA with a length of 46,325 bp and a GC content of 37.8%. A total of 87 open reading frames (ORFs) are predicted in the complete genome of phage vB_AbaP_CV1, among which 27 encode proteins with known functions, including 9 proteins related to DNA replication and transcription, 13 structural proteins, 3 regulatory proteins, 1 host lysis protein, and 1 other protein. The remaining ORFs encode hypothetical proteins. The circular genome map is shown in the figure (**Figure 3A**). In addition, comparative analysis using VFDB and ARDB databases shows that phage vB_AbaP_CV1 does not carry any antibiotic resistance genes or virulence genes, supporting its potential safety *in vivo*. Phylogenetic analysis based on the sequence of the terminase large subunit indicates that vB_AbaP_CV1 has a closer genetic distance with Acinetobacter baumannii phage LZ35 and belongs to the genus Obolenskvirus, class Caudoviricetes, phylum Uroviricota (**Figure 3B**). The anti-biofilm effects of the phage vB_AbaP_CV1 are shown in **Supplementary Figure 2**. The sequence number of vB_AbaP_CV1 has been uploaded to the NCBI database as PX968305.

### 3.5 Synergistic antibacterial activity of vB_AbaP_CV1 combined with polymyxin B *in vitro*

**Figure 4:**
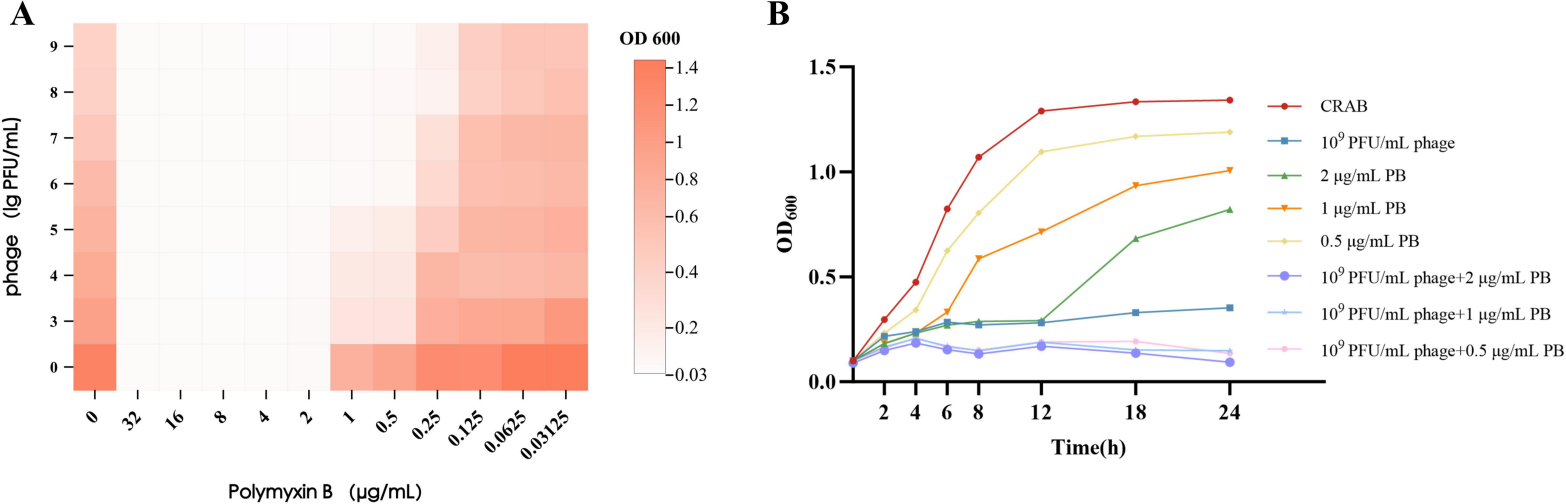
Bactericidal effect of phage combined with polymyxin B. **(A)** Growth heatmap after 16 h treatment with phage combined with polymyxin B; **(B)**Growth kinetics of different treatment groups.

The synergistic effect of vB_AbaP_CV1 and polymyxin B was evaluated by the checkerboard assay. The minimum inhibitory concentration (MIC) of polymyxin B alone was 2 μg/mL.When combined with vB_AbaP_CV1, the MIC of polymyxin B was reduced to 0.5 μg/mL (**Figure 4**). The fractional inhibitory concentration index (FICI) was 0.25(FICI = 0 + 0.5).Since the FICI was 0.25 (< 0.5), the combination of vB_AbaP_CV1 and polymyxin B showed a synergistic antibacterial effect against the tested strain.

To investigate the combined efficacy of vB_AbaP_CV1 and polymyxin B (PB), we monitored the bacterial killing dynamics over 24 hours. Single-agent polymyxin B (0.5, 1, and 2 μg/mL) initially suppressed bacterial growth, but a rebound of resistance was observed after 16 hours, with a rapid increase in OD_600_ values. Single-agent vB_AbaP_CV1 therapy (10^9^ PFU/mL) effectively sustained the inhibition of CRAB growth throughout the 24-hour period, with no noticeable bacterial rebound. Combined vB_AbaP_CV1 and polymyxin B therapy (10^9^ PFU/mL phage + 0.5/1/2 μg/mL PB), the OD_600_ values remained below 0.15 throughout the 24-hour period, indicating inhibition of bacterial growth.

### 3.6 Antibiofilm effect of phage combined with polymyxin B

**Figure 5:**
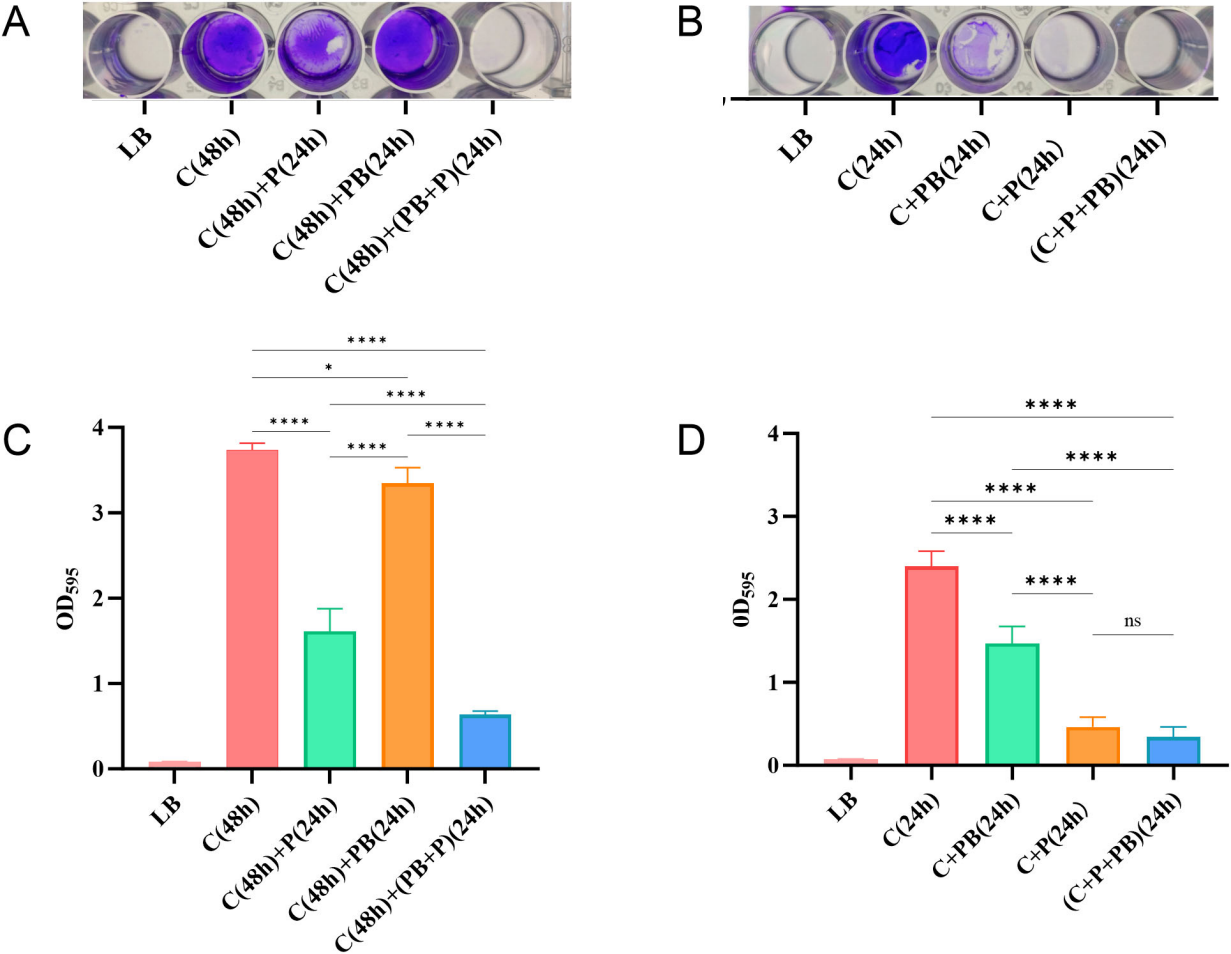
Antibiofilm effect of phage combined with polymyxin B. **(A, B)** Biofilm crystal violet staining. **(C, D)** OD_595_ absorbance of crystal violet-stained biofilms. C(48h):CRAB-1–48 h culture without treatment; C(48h)+P(24h): 48 h culture + 24 h phage treatment; C(48h)+PB(24h): 48 h culture + 24 h polymyxin B treatment; C(48h)+(PB+P)(24h): 48 h culture + 24 h combined treatment with phage and polymyxin B;C(24h): 24 h culture without treatment; C+PB(24h): 24 h culture with concurrent polymyxin B treatment; C+P(24h): 24 h culture with concurrent phage treatment; (C+P+PB)(24h): 24 h culture with concurrent combined treatment of phage and polymyxin B *, p<0.1; ****, p<0.0001.

Based on the results of checkerboard assays and growth curves, we further evaluated the effect of vB_AbaP_CV1 (10^9^ PFU/mL) in combination with polymyxin B (PB, 2 μg/mL) on CRAB biofilms. In the mature biofilm eradication experiment, 48-hour mature CRAB biofilms were established and then treated with single agents or the combination for 24 hours (**Figure 5A**). The results showed that vB_AbaP_CV1 alone exhibited significantly stronger eradication efficacy than PB alone, and the combination treatment resulted in an extremely significant reduction in biofilm biomass compared to the untreated control (****P < 0.0001) (**Figure 5C**). In the biofilm formation inhibition experiment, assessed by co-culturing bacteria with phage or PB (**Figure 5B**). vB_AbaP_CV1 alone significantly inhibited biofilm formation compared to PB alone (****P < 0.0001), and the combination treatment further enhanced this inhibitory effect, which was significantly superior to either monotherapy, demonstrating the potential to efficiently eradicate mature biofilms and synergistically inhibit biofilm formation (**Figure 5D**).

### 3.7 Therapeutic efficacy of phage in CRAB bloodstream infection model

**Figure 6:**
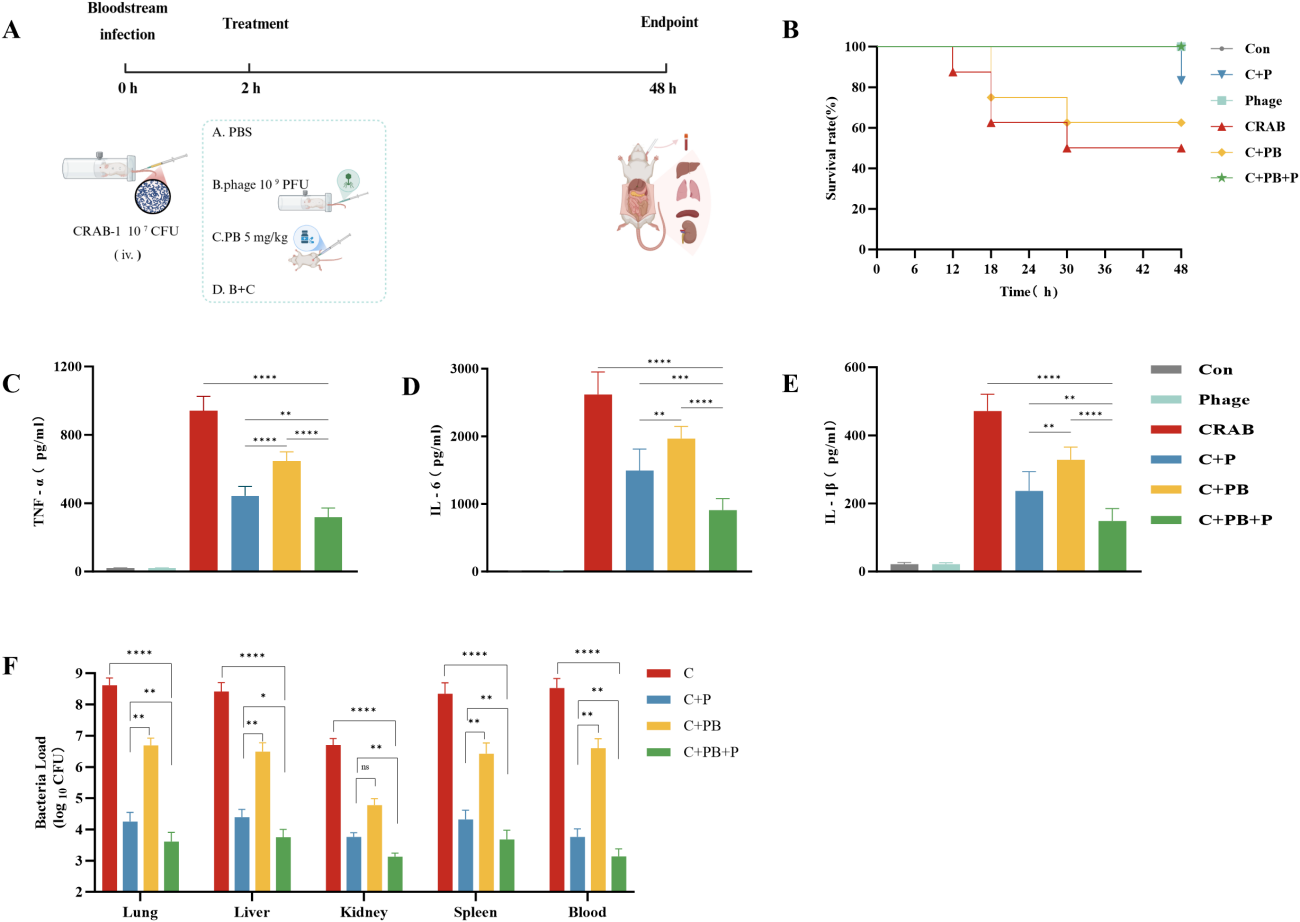
Therapeutic efficacy of vB_AbaP_CV1 combined with PB in a murine model of CRAB-1 bloodstream infection. **(A)** An overview of the *in vivo* experiment. **(B)** Survival curves of mice after CRAB-1 infection and post-1 h treated with vB_AbaP_CV1 and PB. **(C)** TNF-α level in serum **(D)** IL-6 level **(E)** IL-1β level **(F)** Bacterial Load in lung, liver, kidney, spleen and blood. Each group initially contained eight mice (n = 8) *, p<0.1; **, p<0.01; ***, p<0.001; ****, p<0.0001.

In this study, intravenous injection of 1×10^7^ CFU of CRAB-1 resulted in a 50% mortality rate in untreated mice (CRAB group) within 48 hours. When treatment was initiated at 2 hours post-infection, monotherapy with vB_AbaP_CV1, monotherapy with PB, and combination therapy with vB_AbaP_CV1 and PB all significantly improved the survival rate of mice compared to the CRAB group (**Figure 6A**). Specifically, the survival rate of the Phage group (87.5%) was significantly higher than that of the PB group (62.5%), while the combination therapy with vB_AbaP_CV1 and PB group achieved a 100% survival rate (**Figure 6B**). The Mouse Sepsis Score (MSS) was used to assess activity, fur status, respiration, and consciousness every 6 hours from 6 to 48 hours post-infection. The CRAB group showed a continuous increase in clinical scores, peaking at >21 at 48 hours. The combination therapy group exhibited the most significant clinical improvement. The PB monotherapy group had significantly higher scores than the vB_AbaP_CV1 group from 18 hours post-infection (**Supplementary Figure 2**).

At 48 hours post-infection, measurement of pro-inflammatory cytokines in serum revealed that all treatment groups exhibited significantly reduced levels of TNF-α, IL-6, and IL-1β compared to the CRAB group (**Figures 6C, D, E**). Among these, the combination therapy with vB_AbaP_CV1 and PB group showed the most pronounced reduction in inflammatory cytokine levels (P < 0.0001). Additionally, the vB_AbaP_CV1 group displayed significantly lower inflammatory cytokine levels than the PB group (P < 0.001).

Quantitative analysis of bacterial loads in the liver, kidney, lung, spleen, and blood demonstrated that all treatment groups led to a significant reduction in bacterial burden compared to the CRAB group (**Figure 6F**). The combination therapy with vB_AbaP_CV1 and PB group exhibited the greatest reduction in bacterial load, with a decrease of 4 to 5 orders of magnitude (P < 0.0001). With the exception of the kidney, the vB_AbaP_CV1 group was more effective at clearing bacteria in all other tissues and blood than the PB group, achieving an additional reduction of approximately 2 orders of magnitude in bacterial load.

**Figure 7:**
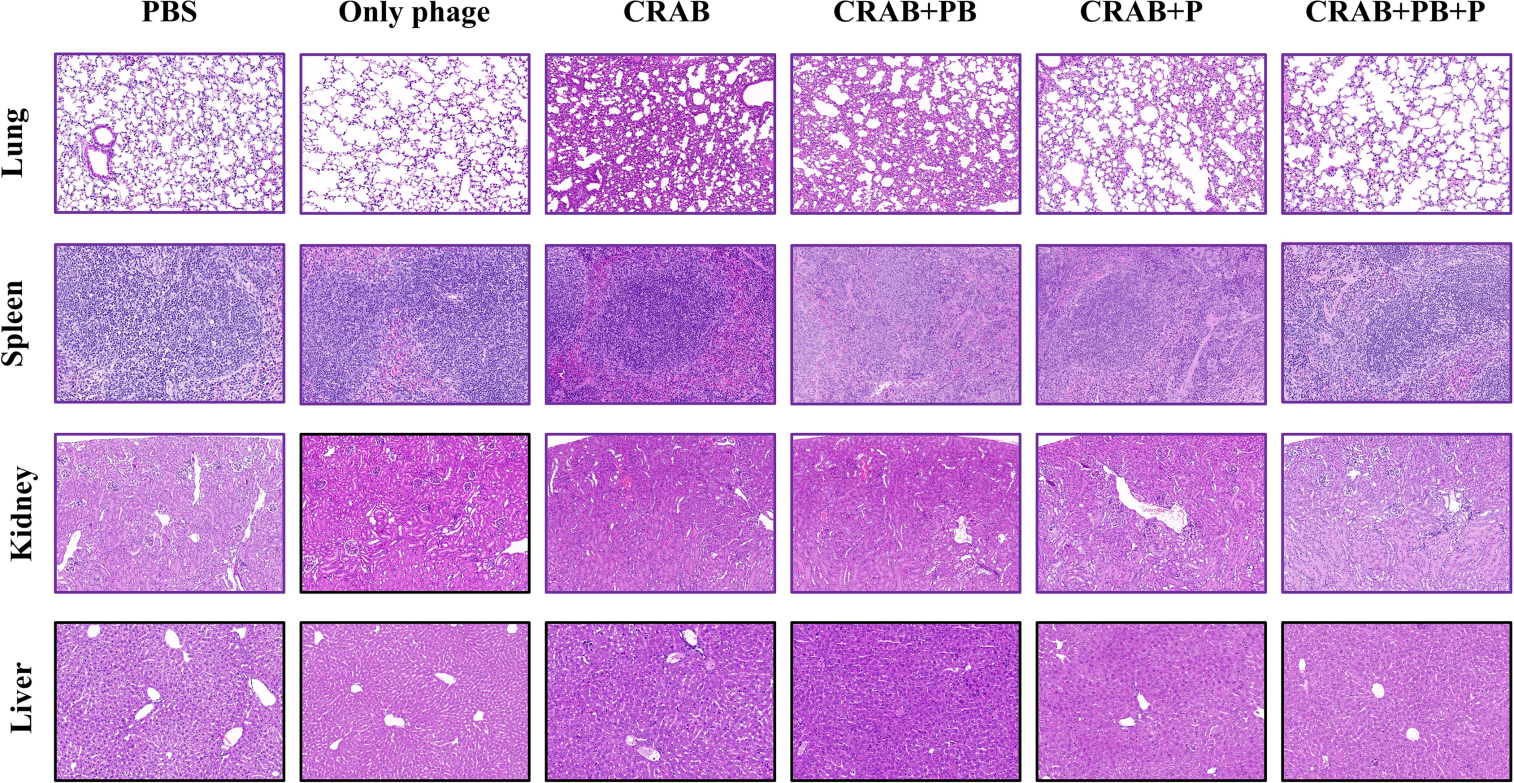
Histopathological analysis of lung, spleen, liver and kidney tissues in mice after different treatments.

HE staining results showed that compared with the PBS control group, mice in the CRAB-only infection group (CRAB group) exhibited severe histopathological damage in four major organs: lung, spleen, kidney, and liver (**Figure 7**). Specifically, the lungs displayed alveolar structural destruction, inflammatory infiltration, and parenchymal consolidation; the spleen showed white pulp hyperplasia and red pulp congestion and edema; the kidneys exhibited renal tubular epithelial cell degeneration, necrosis, and interstitial edema; and the liver presented with disrupted hepatic lobule structure, hepatocyte swelling, and inflammatory cell infiltration.

Treatment with vB_AbaP_CV1 alone (Only phage group) did not induce obvious tissue toxicity, and the histological morphology was comparable to that of the PBS group. Compared with the CRAB group, treatment with either polymyxin B alone (CRAB+PB group) or phage alone (CRAB+P group) partially alleviated pathological lesions in all organs, but the protective effect of polymyxin B alone was weaker than that of phage alone. In contrast, the combination of phage and polymyxin B (CRAB+PB+P group) resulted in histological morphology closest to that of the PBS group, with significant improvement in pathological changes and markedly reduced inflammatory infiltration across all organs, indicating the best tissue protective effect. These findings suggest that the combined application of phage and polymyxin B can effectively mitigate CRAB infection-induced multiple organ injury.

## 4 Discussion

Acinetobacter baumannii is a major cause of healthcare-associated infections, and carbapenem-resistant *A. baumannii* (CRAB) bloodstream infections are associated with particularly high mortality (WHObacterial priority pathogens list, 2024: bacterial pathogens of public health importance to guide research, development and strategies to prevent and control antimicrobial resistance, 2024). Previous studies have reported mortality rates ranging from 40% to over 70% in critically ill populations (Playford et al., 2007). In our single-center cohort, the in-hospital mortality of CRAB patients reached 82.5%, markedly higher than that of CSAB patients, underscoring the severe clinical burden of carbapenem resistance.

The global rise of antimicrobial resistance has significantly limited therapeutic options for CRAB (Tamma et al., 2024). Once carbapenem resistance develops, isolates frequently exhibit multidrug-resistant phenotypes, leaving polymyxins, tigecycline, or combination regimens as last-resort treatments (Pompilio et al., 2021). Bacteriophage therapy has re-emerged as a promising precision antibacterial strategy due to its high host specificity and self-amplifying nature. In the present study, we isolated and characterized a CRAB-targeting phage, vB_AbaP_CV1, which lysed 72% of tested CRAB clinical isolates and exhibited favorable environmental stability without carrying virulence or antibiotic resistance genes. These findings are consistent with recent reports highlighting the therapeutic potential of lytic phages against multidrug-resistant *A. baumannii*. However, susceptibility testing in CSAB strains was not performed in the present study. Therefore, the relationship between carbapenem susceptibility and phage responsiveness remains unclear. Although phage infection is generally mediated by specific bacterial surface receptors rather than antibiotic resistance phenotypes, whether receptor alterations associated with carbapenem resistance influence phage susceptibility requires further investigation.

Polymyxin B remains a key agent for CRAB treatment but is limited by nephrotoxicity and a narrow therapeutic window. Growing evidence suggests that phage–antibiotic combination strategies may enhance bacterial killing and suppress resistance emergence (Choudhary and Shariff, 2025; Kubin et al., 2025). In our study, checkerboard and time-kill analyses demonstrated clear synergy between vB_AbaP_CV1 and polymyxin B (FICI = 0.25), with a 75% reduction in polymyxin B MIC and effective suppression of resistant subpopulations. These results support the concept that phage–antibiotic combinations may restore antibiotic susceptibility and reduce required dosages, potentially improving therapeutic safety.

Biofilm formation further complicates CRAB infections, as mature biofilms significantly impair antibiotic penetration and contribute to persistent infection (Chegini et al., 2020; Mukhopadhyay et al., 2024). Consistent with previous observations that CRAB strains exhibit strong biofilm-forming capacity, we found that vB_AbaP_CV1 alone could inhibit biofilm formation and disrupt established biofilms, while polymyxin B showed limited activity against mature biofilms. The combination therapy demonstrated the strongest biofilm-disrupting and inhibitory effects, suggesting a complementary mechanism of action.

*In vivo* validation represents a critical preclinical step for translational development. Genomic sequencing confirmed that vB_AbaP_CV1 lacks lysogenic, virulence, and antibiotic resistance genes, supporting its genetic safety profile. In the murine CRAB bloodstream infection model, phage monotherapy significantly improved survival and clinical parameters, while combination therapy achieved the most pronounced therapeutic benefit. The limited durability of polymyxin B monotherapy may reflect suboptimal pharmacokinetics following a single dose. Collectively, these findings align with emerging evidence that phage–antibiotic combinations can enhance therapeutic efficacy against multidrug-resistant pathogens.

Despite these encouraging findings, several limitations should be acknowledged. This was a single-center study, and regional epidemiological variability cannot be excluded. Larger, multicenter studies are required to better define the epidemiology and risk factors of CRAB bloodstream infections. In addition, mechanistic studies are needed to elucidate the molecular basis of the observed phage–antibiotic synergy. Optimization of dosing strategies, treatment timing, and administration regimens will be essential for clinical translation. Further investigation of phage interactions with additional antibiotic classes is also warranted.

## 5 Conclusion

In conclusion, CRAB bloodstream infections are associated with high mortality and poor prognosis, posing a significant clinical challenge. Phage vB_AbaP_CV1 was identified as a lytic phage with activity against clinically isolated CRAB strains. *In vitro* experiments verified the synergistic effect between vB_AbaP_CV1 and polymyxin B. Preclinical evaluation in BALB/c mice confirmed its therapeutic potential, suggesting that vB_AbaP_CV1 may serve as a viable alternative to antibiotics for the treatment of CRAB infections in clinical applications.

## Data Availability

The sequences of the bacteriophage characterized in this study were deposited in GenBank under the accession number PX968305 (https://www.ncbi.nlm.nih.gov/nuccore/PX968305). All other original data generated or analyzed during this study are available from the corresponding author upon reasonable request.
