# Supplementary material for: Carbapenem-resistant Acinetobacter baumannii bloodstream infections and specific phages isolation, analysis and application
Source: Front Cell Infect Microbiol. 2026 Apr 1;16:1774993. doi: 10.3389/fcimb.2026.1774993 (PMC13079707; doi:10.3389/fcimb.2026.1774993)

Supporting information

Carbapenem-Resistant Acinetobacter Baumannii (CRAB) Bloodstream Infections And Specific Phages isolation、analysis and application

# Lu Wang^1^; Fuhua Wang^1^; Zhiyong Yuan^1^; Ying Liu^1^; Yajun Jing^1^; Jinyan Xing^1^

^1^Department of Critical Care Medicine, the Affiliated Hospital of Qingdao University, 16 Jiangsu Road, Qingdao, Shandong Province, China，266000

*** Correspondence:**Jinyan Xing, PhD. [xingjy@qdu.edu.cn](mailto:xingjy@qdu.edu.cn)

**Table S1** Bioinformatics Analysis of the CRAB-1 Strain

| **Stain** | **Souce** | **gMLST** | **Antibiotic resistance genes identified by whole genome sequencing** |
| --- | --- | --- | --- |
| CRAB-1 | blood | ST369 | blaOXA-23,blaOXA-51, blaADC; gyrA, parC, parE mutations; ant(3''), armA; RND, MFS, ABC efflux pumps; cat; folP mutation; rpoB mutation |

**Table S2** CRAB-1 antimicrobial susceptibility testing

| **Antibiotic** | **MIC (μg/mL)** | **Interpretation** |
| --- | --- | --- |
| Cefoperazone/Sulbactam | ＞64 | R |
| Ampicillin/Sulbactam | ≥32 | R |
| Piperacillin/Tazobactam | ＞128 | R |
| Meropenem | ＞16 | R |
| Imipenem | ＞32 | R |
| Ceftazidime | ＞64 | R |
| Cefepime | ＞64 | R |
| Ciprofloxacin | ＞4 | R |
| Levofloxacin | ＞8 | R |
| Amikacin | ＞16 | R |
| Gentamicin | ＞16 | R |
| Tobramycin | ＞16 | R |
| Minocycline | ≤0.5 | **S** |
| Polymyxin B | 2 | **S** |
| Tigecycline | ≤0.5 | **S** |

**Table S3 Host range of phage vB_AbaP_CV1**

| **Stains** | **Date of isolation** | **Source** | **Susceptibility** |
| --- | --- | --- | --- |
| CR-AB209055 | 2020-09-24 | blood | **+** |
| CR-AB219068 | 2021-10-13 | sputum | - |
| CR-AB229018 | 2022-02-04 | blood | **+** |
| CR-AB229058 | 2022-06-16 | blood | - |
| CR-AB229081 | 2022-07-29 | blood | **+** |
| CR-AB239100 | 2023-01-07 | blood | **+** |
| CR-AB239109 | 20232-01-17 | sputum | **+** |
| CR-AB239184 | 2023-11-20 | blood | **+** |
| CR-AB249023 | 2024-01-18 | sputum | **+** |
| CR-AB249042 | 2024-03-02 | sputum | - |
| CR-AB249045 | 2024-03-15 | blood | **+** |
| CR-AB249057 | 2024-04-09 | blood | **+** |
| CR-AB249079 | 2024-05-28 | sputum | **+** |
| CR-AB249114 | 2024-06-05 | BALF | - |
| CR-AB249159 | 2024-06-29 | urine | **+** |
| CR-AB249171 | 2024-08-06 | blood | **+** |
| CRAB249183 | 2024-08-16 | blood | - |
| CR-AB249201 | 2024-09-21 | sputum | **+** |
| CR-AB249214 | 2024-10-30 | BALF | **+** |
| CRAB259010 | 2025-01-18 | blood | - |
| CR-AB259012 | 2025-02-01 | blood | **+** |
| CR-AB259034 | 2025-02-19 | BALF | - |
| CR-AB259039 | 2025-03-01 | BALF | **+** |
| CR-AB259051 | 2025-03-29 | blood | **+** |
| CR-AB259066 | 2025-04-06 | blood | **+** |
| K. Pneumoniae-1 | 2025-01-10 | sputum | - |
| K. Pneumoniae-2 | 2025-02-23 | blood | - |
| K. Pneumoniae-3 | 2025-03-14 | blood | - |
| K. Pneumoniae-4 | 2025-03-28 | BALF | - |
| K. Pneumoniae-5 | 2025-04-02 | blood | - |
| E.coli -1 | 2025-02-18 | blood | - |
| E.coli -2 | 2025-03-01 | sputum | - |
| E.coli -3 | 2025-04-11 | BALF | - |
| E.coli -4 | 2025-04-17 | sputum | - |
| E.coli -5 | 2025-04-30 | blood | - |
| P.aeruginosa -1 | 2025-02-10 | blood | - |
| P.aeruginosa -2 | 2025-02-28 | sputum | - |
| P.aeruginosa -3 | 2025-03-01 | sputum | - |
| P.aeruginosa -4 | 2025-04-01 | BALF | - |
| P.aeruginosa -5 | 2025-04-06 | skin | - |

Figure S1 Survival curves of mice after tail vein injection of CRAB-1 at different concentrations


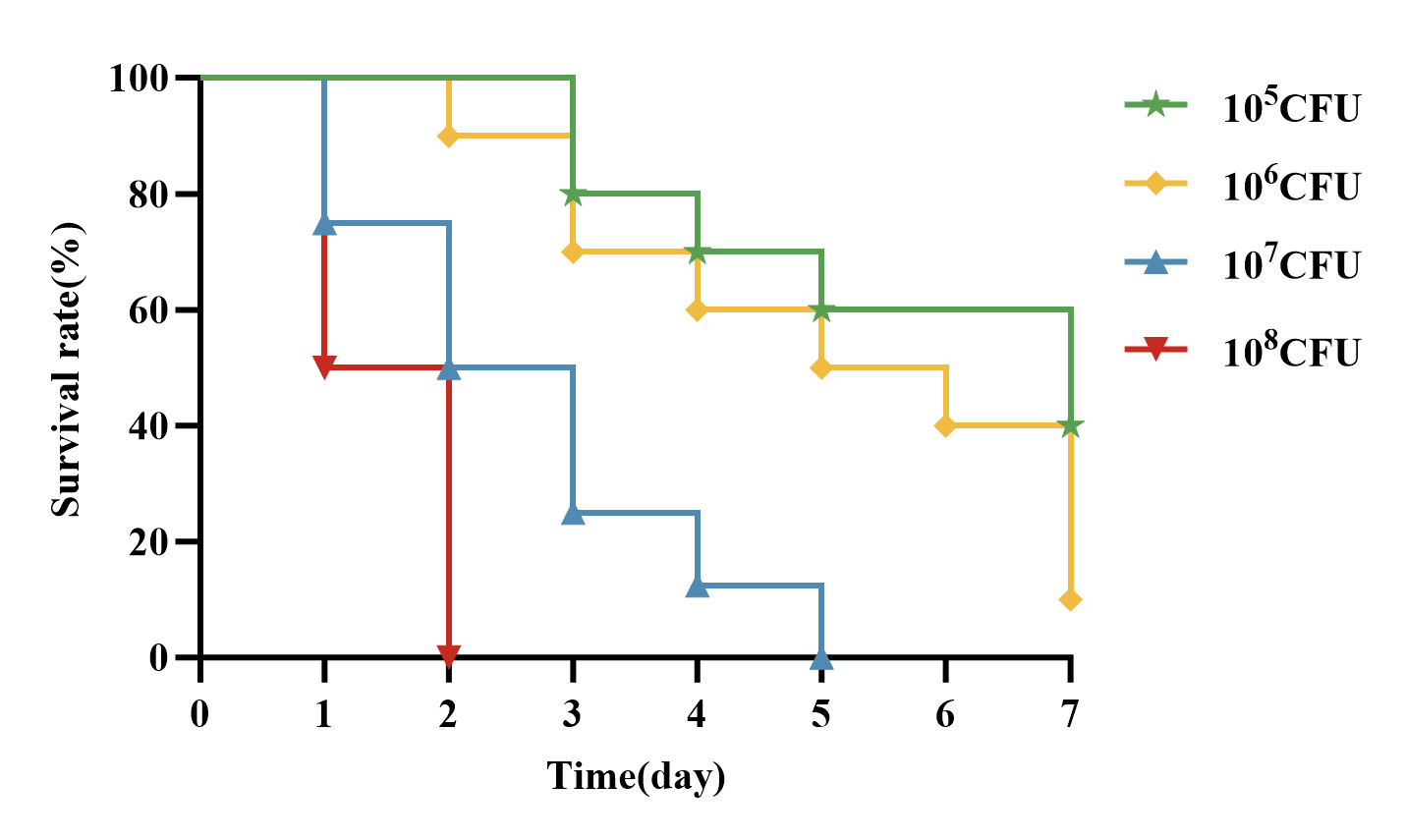


Figure S2 Murine Sepsis Score (MSS) of mice


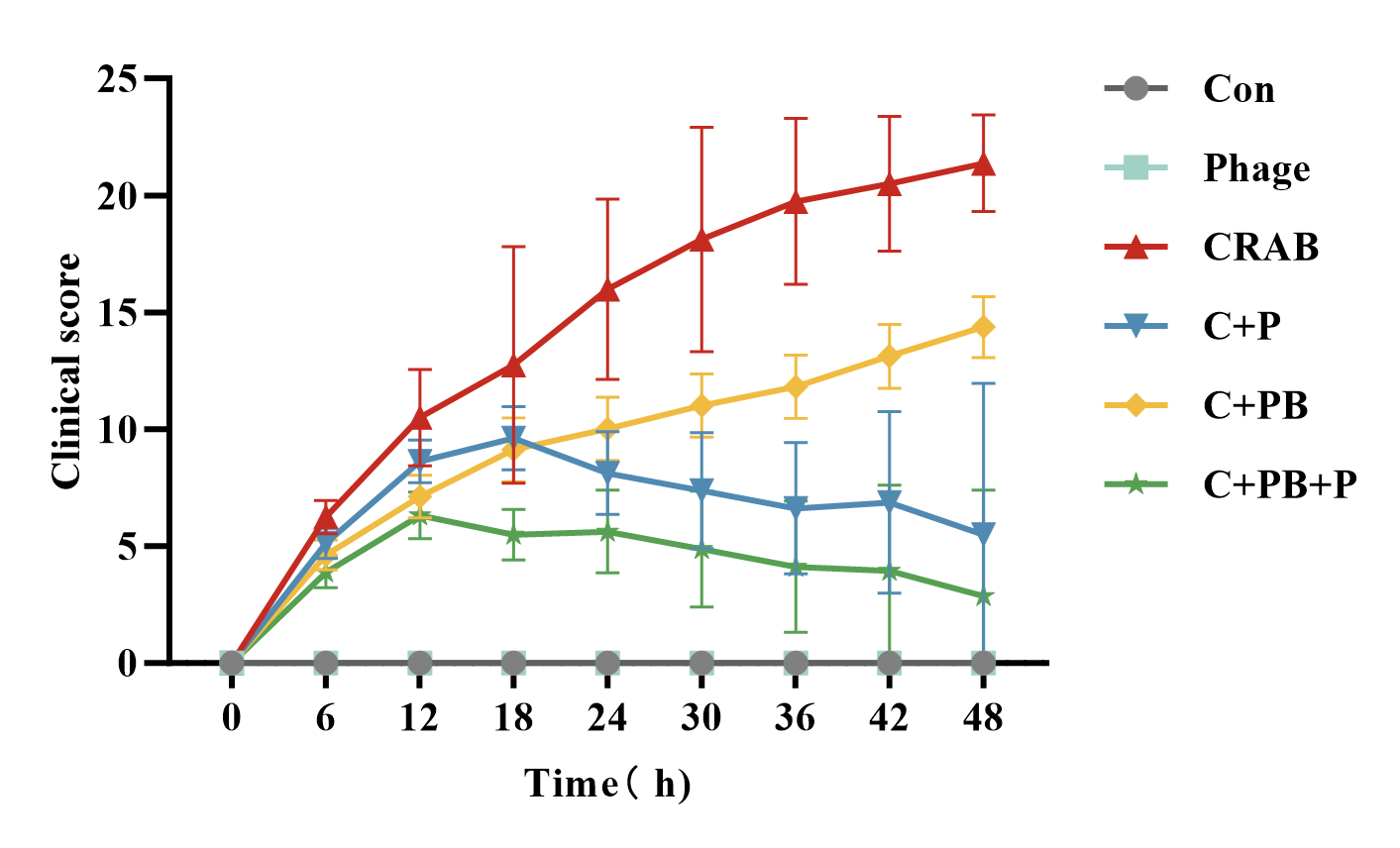

Supplement: Supplementary file 1 [file Table1.docx]
